# Supplementary material for: Enhancing Early GI Disease Detection with Spectral Visualization and Deep Learning
Source: Bioengineering (Basel). 2025 Jul 30;12(8):828. doi: 10.3390/bioengineering12080828 (PMC12383988; doi:10.3390/bioengineering12080828)
Supplement: Supplementary file 1 [file bioengineering-12-00828-s001.zip › bioengineering-3770192-supplementary.pdf]

bioengineering

MDPI

Supplementary

Enhancing Early GI Disease Detection with Spectral Visualization and Deep Learning: Supplementary Material

Tsung-Jung Tsai [1], Kun-Hua Lee [2], Chu-Kuang Chou [1,3], Riya Karmakar [4], Arvind Mukundan [4,5], Tsung-Hsien Chen [6], Devansh Gupta [7], Gargi Ghosh [8], Tao-Yuan Liu [9,10,*] and Hsiang-Chen Wang [4,11,12,*]

[1] Division of Gastroenterology and Hepatology, Department of Internal Medicine, Ditmanson Medical Foundation Chia-Yi Christian Hospital, Chia-Yi 60002, Taiwan
[2] Department of Trauma, Changhua Christian Hospital, Changhua, No. 135, Nanxiao St., Changhua City, Changhua County 50006, Taiwan
[3] Obesity Center, Ditmanson Medical Foundation Chia-Yi Christian Hospital, Chia-Yi 60002, Taiwan
[4] Department of Mechanical Engineering, National Chung Cheng University, 168, University Rd., Min Hsiung, Chia-Yi 62102, Taiwan
[5] Department of Biomedical Imaging, Chennai Institute of Technology, Sarathy Nagar, Chennai 600069, Tamil Nadu, India
[6] Department of Internal Medicine, Ditmanson Medical Foundation Chia-Yi Christian Hospital, Chia-Yi 60002, Taiwan
[7] Computer Science and Engineering Department, Thapar Institute of Engineering & Technology, Patiala 147001, Punjab, India
[8] Apex Institute of Technology, Chandigarh University, Ludhiana 140413, Punjab, India
[9] Department of Pediatrics, Kaohsiung Armed Forces General Hospital, 2, Zhongzheng 1st. Rd., Lingya District, Kaohsiung City 80284, Taiwan
[10] Department of Medicine, National Defense Medical University, No.161, Sec. 6, Minquan E. Rd., Neihu District, Taipei City 11490, Taiwan
[11] Department of Medical Research, Dalin Tzu Chi Hospital, Buddhist Tzu Chi Medical Foundation, No. 2, Minsheng Road, Dalin, Chia-yi 62247, Taiwan
[12] Department of Technology Development, Hitspectra Intelligent Technology Co., Ltd., Kaohsiung 80661, Taiwan
* Correspondence: a1969928@gmail.com (T.-Y.L.); hcwang@ccu.edu.tw (H.-C.W.)

Abstract: Timely and accurate diagnosis of gastrointestinal diseases (GID) remains a critical bottleneck in clinical endoscopy, particularly due to the limited contrast and sensitivity of conventional white light imaging (WLI) in detecting early-stage mucosal abnormalities. To overcome this, this research presents SAVE (Spectrum Aided Vision Enhancer) an innovative, software-driven framework that transforms standard WLI into high-fidelity hyperspectral imaging (HSI) and simulated narrow-band imaging (NBI) without any hardware modification. SAVE leverages advanced spectral reconstruction techniques, including Macbeth Color Checker-based calibration, principal component analysis (PCA), and multivariate polynomial regression, achieving a root mean square error (RMSE) of 0.056 and structural similarity index (SSIM) exceeding 90%. Trained and validated on the Kvasir v2 dataset (n = 6,490) using deep learning models like ResNet50, ResNet101, EfficientNet-B2, B5 and EfficientNetV2-B0 were used to assess diagnostic performance across six key GI conditions. Results demonstrated that SAVE enhanced imagery consistently outperformed raw WLI across precision, recall, and F1-score metrics, with EfficientNet-B2 and EfficientNetV2-B0 achieving the highest classification accuracy. Notably, this performance gain was achieved without the need for specialized imaging hardware. These findings highlight SAVE as a transformative solution for augmenting GI diagnostics, with the potential to significantly improve early detection, streamline clinical workflows, and broaden access to advanced imaging especially in resource constrained settings

 https://doi.org/10.3390/bioengineering12080828

Keywords: Gastrointestinal diseases; Endoscopy; Hyperspectral imaging; Narrow-band imaging; Spectrum-aided vision enhancer; Spectral reconstruction; Colour calibration; Deep learning; Image enhancement; Early diagnosis

S1. Resnet Architecture

S1.1 Resnet 50

Figure S1. Confusion Matrix Resnet 50

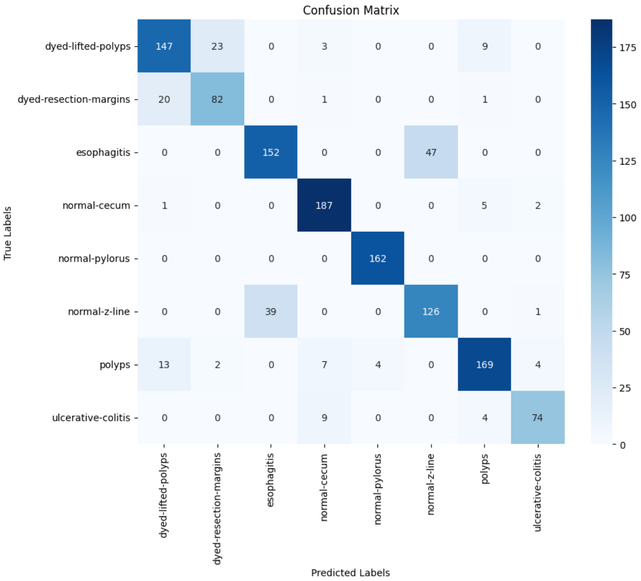

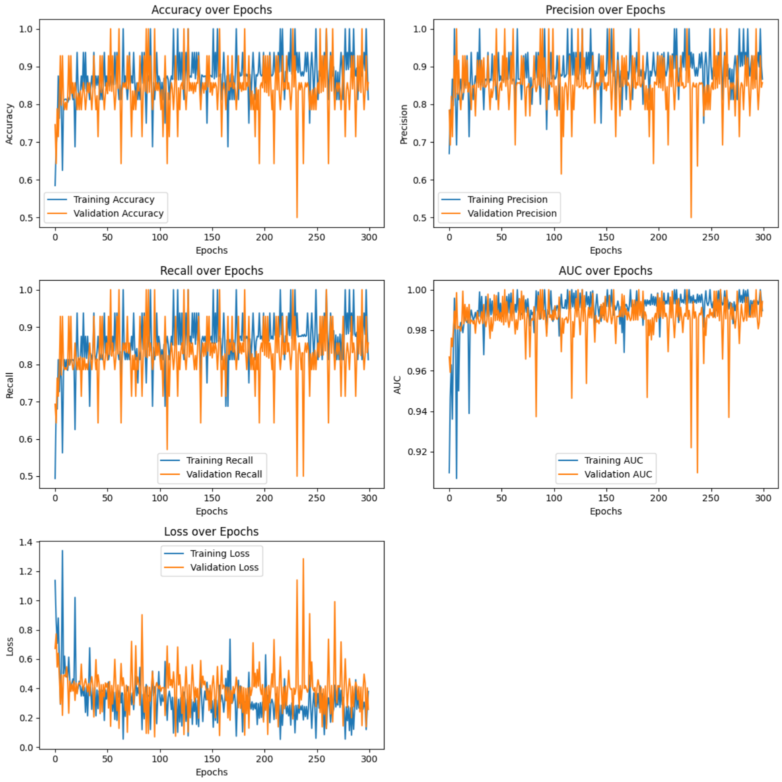

Figure S2. Accuracy, Recall, Precsion, loss, Auc Plots for Resnet 50

S1.2 Resnet 101

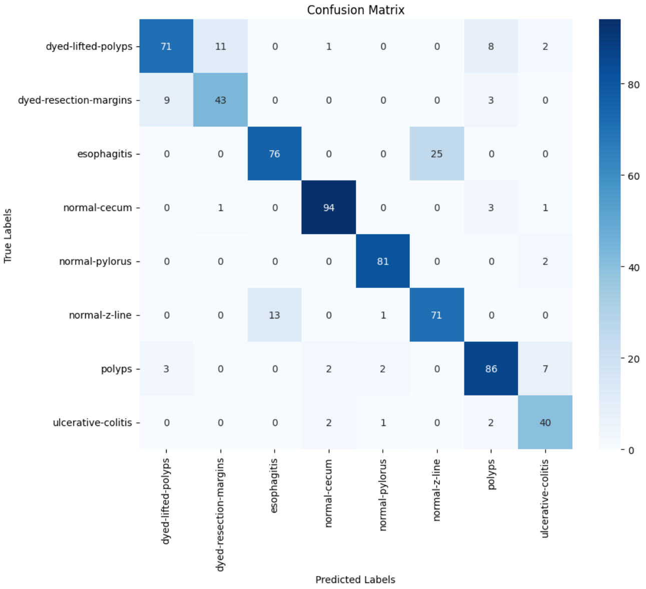

Figure S3. Confusion Matrix Resnet 101

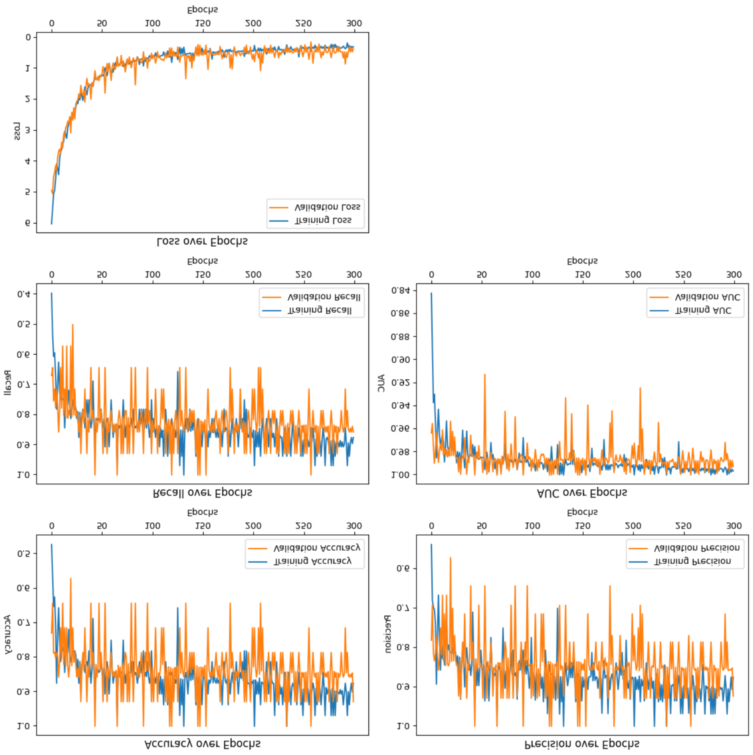

Figure S4. Accuracy, Recall, Precsion, loss, Auc Plots for Resnet 101

Figure S6. YOLOv8 Hyperspectral image training set and validation set loss functions and convergence of precision, recall, and mean precision

S1.3 Efficient Net B2

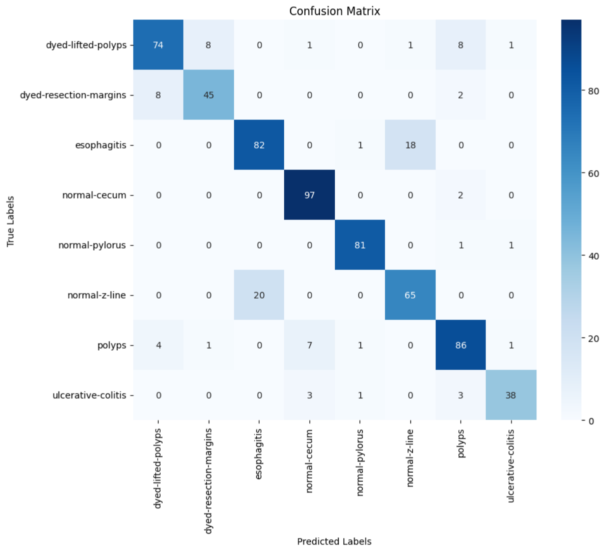

Figure S5. Confusion Matrix Efficient Net B2

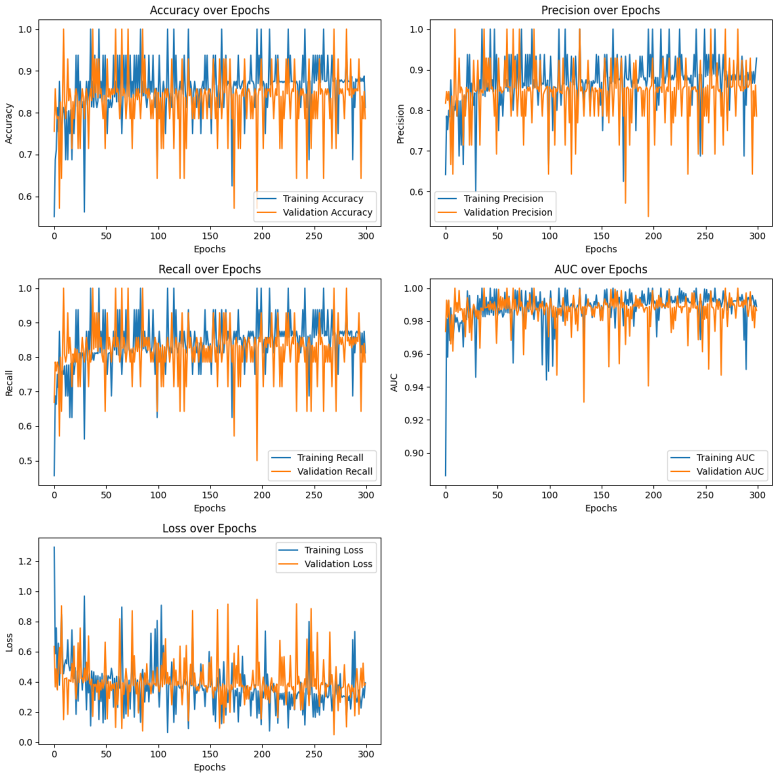

Figure S6. Accuracy, Recall, Precsion, loss, Auc Plots for Efficient Net B2

S1.4 Efficient Net B5

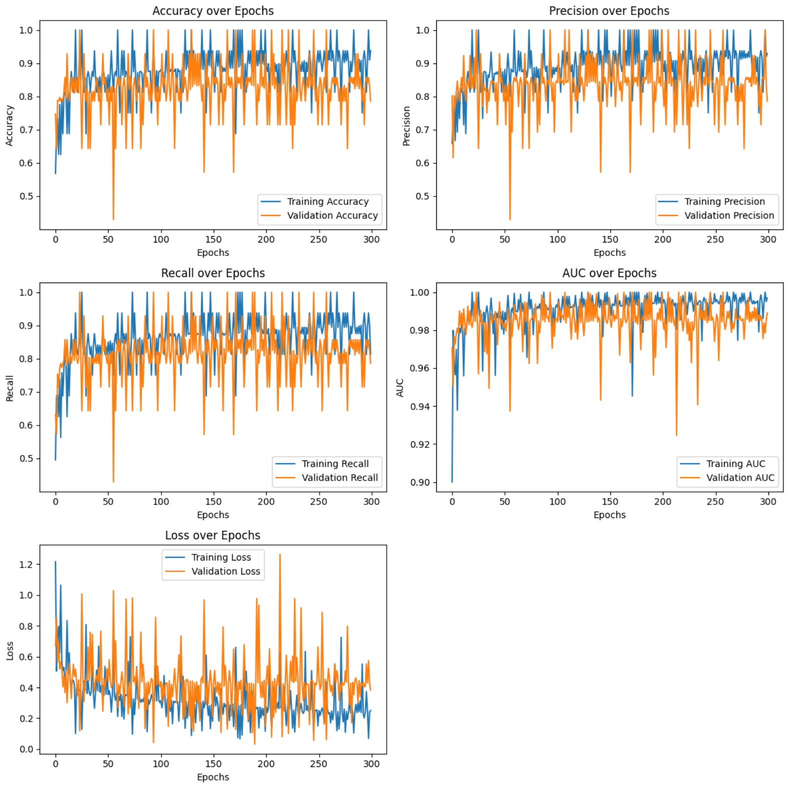

Figure S7. Accuracy, Recall, Precsion, loss, Auc Plots for Efficient Net B5

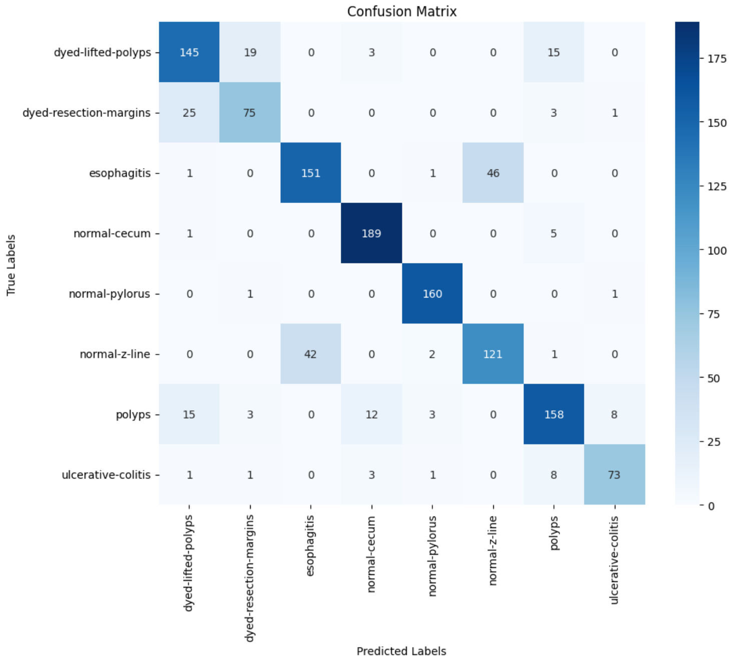

Figure S8. Confusion Matrix Efficient Net B5

S.1.5 Efficient Net V2B0

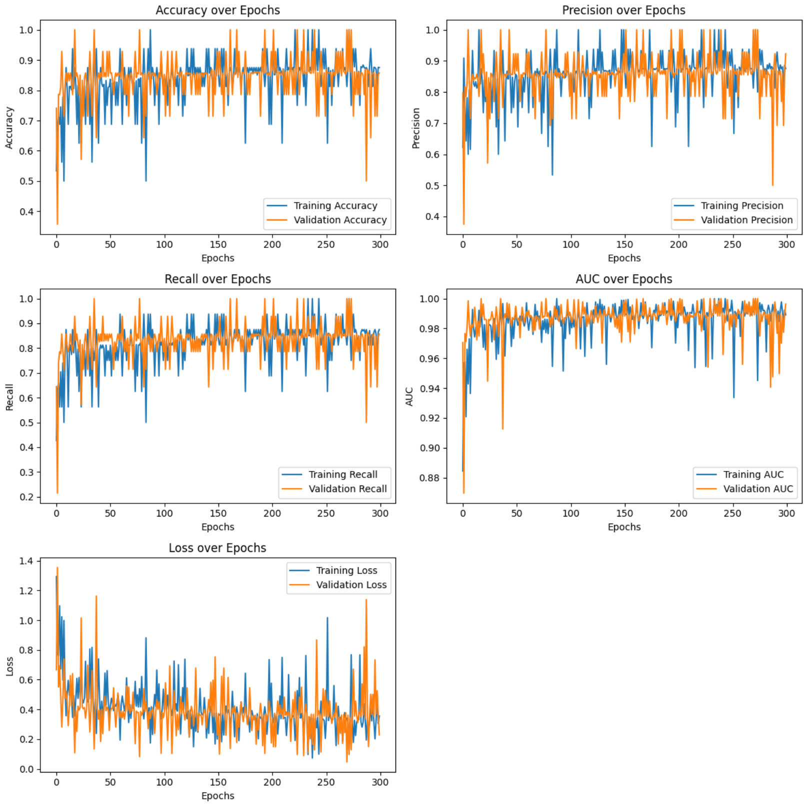

Figure S9. Accuracy, Recall, Precsion, loss, Auc Plots for Efficient Net V2B0

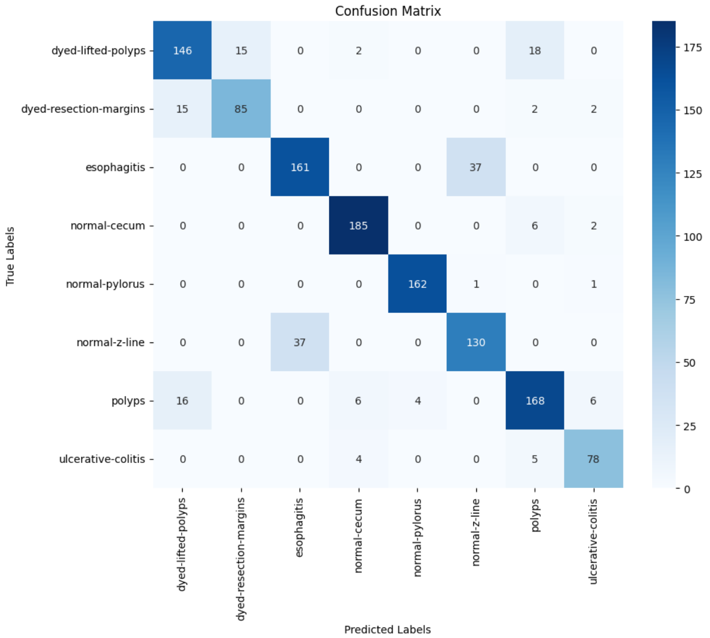

Figure S10. Confusion Matrix Efficient Net V0B0

S.2 SAVE

Figure S11. RMSEs of the XYZ values before and after calibration.

S.no	Before calibration	After calibration	RMSE	SD

	X	Y	Z	X	Y	Z		
1	10.96	9.92	4.63	11.14	9.87	4.26	0.24	0.30
2	38.74	35.80	18.65	38.57	35.94	18.66	0.13	0.08
3	16.62	19.07	24.13	16.48	18.79	24.11	0.18	0.17
4	10.33	12.86	4.62	10.16	13.03	4.85	0.19	0.19
5	24.05	23.87	31.55	24.16	24.07	31.60	0.13	0.08
6	30.12	42.15	32.40	30.10	42.17	32.42	0.02	0.002
7	38.10	30.24	4.28	38.04	30.37	4.22	0.09	0.04
8	11.70	11.47	25.90	11.64	11.37	25.91	0.07	0.02
9	29.01	19.91	9.62	29.20	19.78	9.60	0.13	0.08
10	8.26	6.49	9.63	8.06	6.49	9.86	0.18	0.17
11	34.15	44.06	8.44	34.15	44.02	8.53	0.06	0.01
12	47.99	44.55	6.05	48.05	44.34	6.17	0.15	0.11
13	6.82	5.79	21.07	6.90	5.91	21.00	0.09	0.04
14	14.55	23.55	7.22	14.58	23.51	7.12	0.07	0.02
15	21.08	12.25	3.57	21.01	12.28	3.65	0.06	0.01
16	58.40	60.69	7.54	58.38	60.79	7.42	0.09	0.04
17	28.98	19.54	20.67	28.94	19.52	20.66	0.02	0.002
18	12.81	19.01	28.54	12.84	19.10	28.56	0.05	0.01
19	82.12	88.54	67.20	82.31	88.73	67.51	0.24	0.30
20	54.74	58.92	45.52	54.28	58.40	44.75	0.60	1.89
21	33.08	35.73	27.24	33.26	35.82	27.54	0.21	0.23
22	18.18	19.62	14.94	18.86	20.31	15.62	0.68	2.43
23	9.13	10.01	8.13	8.56	9.26	7.21	0.76	3.04

24	2.87	3.19	2.39	3.10	3.35	2.68	0.23	0.27
Average							0.19	0.39

S.no	Before Camera Calibration	Spectrometer	Chromatic Aberration	After Camera Calibration	Spectrometer	Chromatic Aberration
1			7.08			1.24
2			7.63			0.78
3			16.43			0.86
4			12.45			1.68
5			14.92			0.45
6			10.80			0.05
7			7.47			0.52
8			18.46			0.22
9			13.19			0.62
10			8.09			1.30
11			8.03			0.09
12			6.43			0.58
13			10.32			0.30
14			12.19			0.23
15			13.31			0.17
16			7.00			0.18
17			17.80			0.03
18			22.22			0.19
19			0.00			0.08
20			5.30			0.30
21			9.77			0.42
22			12.71			0.81

23			13.34			2.01
24			3.37			1.96
Average Color Difference			10.76	Average Color Difference		0.63

Figure S 12. The color difference before and after camera calibration

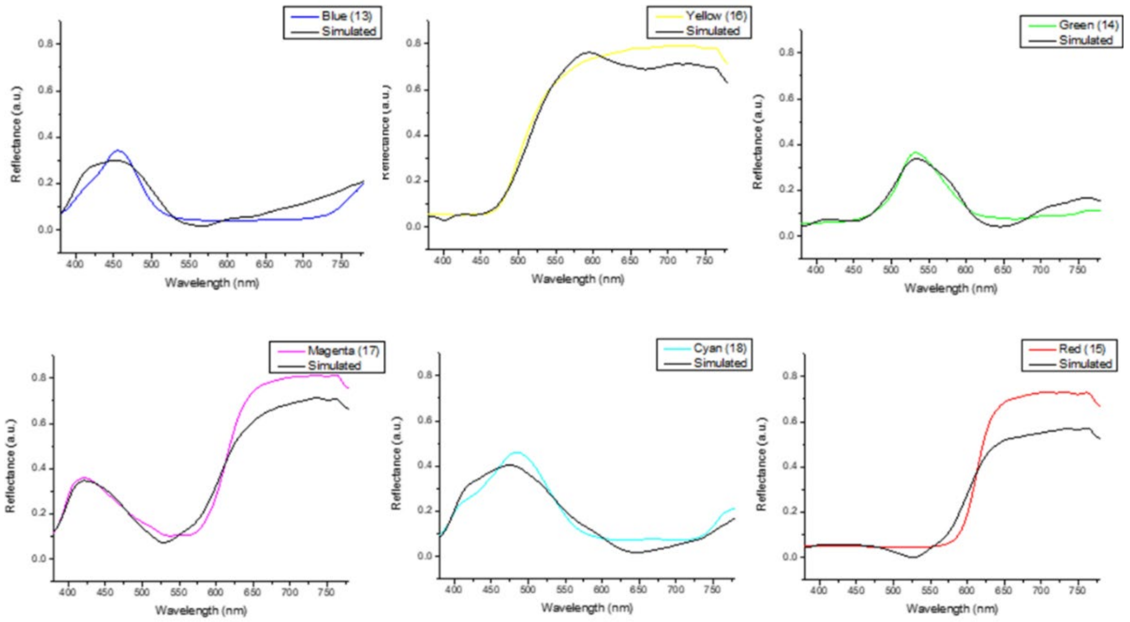

Figure S13. RMSEs between analog and measured spectra of each color block

Measured Color				Simulated Color				Color Difference
L	a	b	Color	L	a	b	Color	
37.61	13.65	24.56		37.64	11.87	22.60		1.32
66.48	14.68	31.10		66.37	15.47	30.58		0.73
50.44	-7.58	-6.44		50.72	-8.16	-6.09		0.75
42.80	-16.14	30.50		42.48	-14.04	31.19		1.50
56.16	5.70	-8.01		55.96	5.89	-8.51		0.43
70.99	-34.14	16.44		71.01	-34.00	15.65		0.41
61.97	32.36	66.76		61.85	33.07	65.32		0.80
40.20	6.07	-27.03		40.27	6.15	-26.67		0.27
51.59	46.04	27.52		51.72	44.60	27.21		0.52
30.62	18.70	-9.45		30.52	20.85	-8.70		1.46
72.24	-24.91	66.55		72.23	-25.21	66.79		0.13
72.46	17.04	75.67		72.59	16.33	76.17		0.49
29.18	13.90	-37.66		28.70	15.23	-38.14		0.81
55.59	-40.93	42.88		55.56	-41.82	42.54		0.42
41.66	53.78	34.95		41.60	54.34	34.25		0.51
82.26	1.48	87.73		82.21	1.96	87.67		0.27
51.29	46.36	1.08		51.30	46.12	0.95		0.10
50.80	-31.41	-12.85		50.66	-30.83	-13.20		0.42
95.47	-3.88	21.64		95.38	-3.70	22.54		0.52
80.96	-3.08	18.47		81.32	-3.39	17.74		0.63
66.38	-2.74	15.56		66.33	-3.33	15.44		0.69
52.18	-2.26	12.86		51.36	-2.59	12.85		0.91
36.47	-2.05	9.55		37.78	-3.18	8.64		2.00
21.40	-1.45	6.28		20.70	-2.87	7.50		2.02
							Average Color Difference	0. 75

Figure S14 LAB values of the simulated and observed colors.

S3. Evaluation of SAVE using VCE against WLI and NBI Olympus Endoscope

NBI is an imaging technique for endoscopic diagnostic medical tests, where light of specific blue and green wavelengths is used to enhance the detail of certain aspects of the surface of the mucosa. A special filter is electronically activated by a switch in the endoscope leading to the use of ambient light of wavelengths of 415 nm (blue) and 540 nm (green). Because the peak light absorption of hemoglobin occurs at these wavelengths, blood vessels will appear very dark, allowing for their improved visibility and the improved identification of other surface structures. However, as shown in Figure S15, the Olympus endoscope's NBI images do not only contain the red and blue colors in it, rather it also contain hues of brown, orange and red in it.

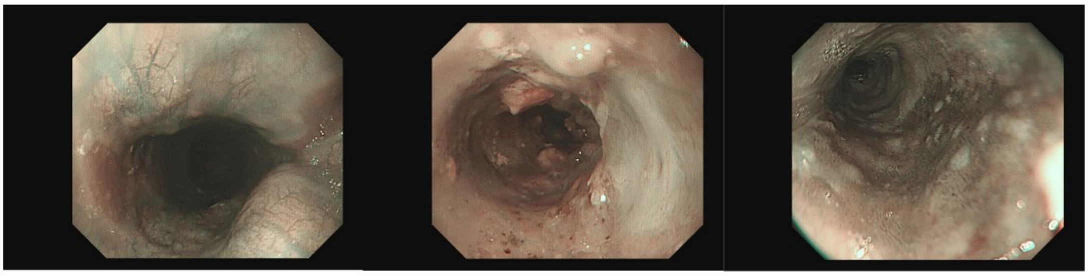

(a) (b) (c)

Figure S15. Shows the Olympus Endoscope's NBI images with orange, red, and brown hues.

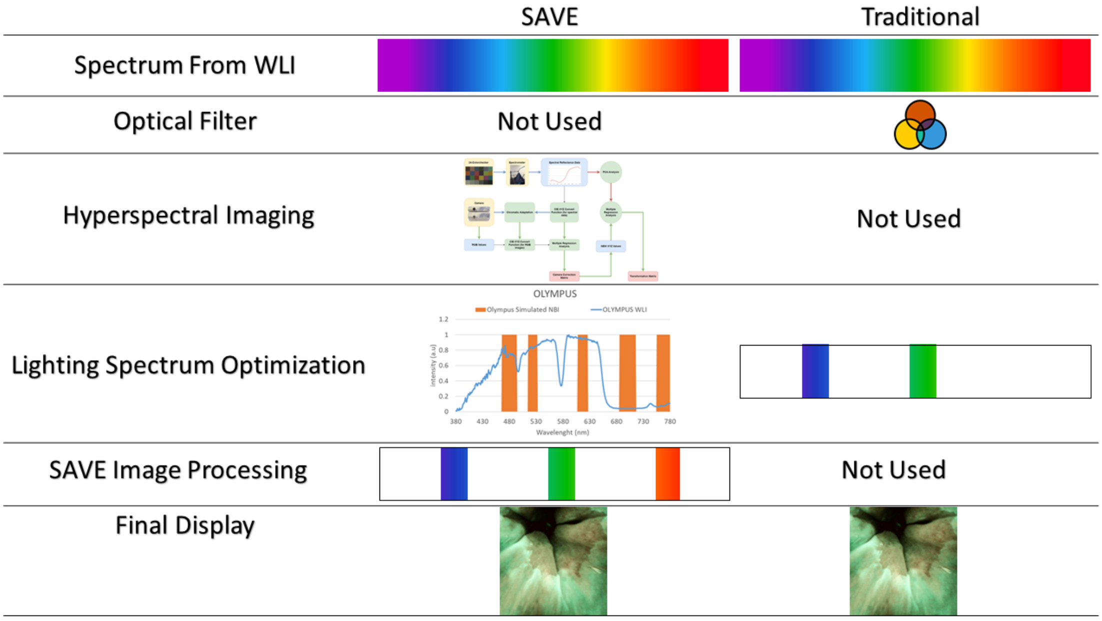

Figure S16. Shows the difference between Traditional and SAVE imaging modalities

The valuation of the SAVE algorithm is comprehensively assessed through its performance metrics, specifically the Structural Similarity Index Metric (SSIM), pixel signal to noise ratio (PSNR) and entropy, which provide quantitative insights into the algorithm's effectiveness in image reproduction. At first SSIM between the simulated and the real WLI of the Olympus endoscope can be compared with the SAVE image from the HSI conversion algorithm developed in this study. SSIM can be defined as the amount of similarities between the simulated and the real image. The values range between 0% to 100% where 100% means the images are completely the same and 0% means the images are completely different. It is measured for the images that are reconstructed from a base image. Therefore, this parameter fits our criteria of comparison. Similarly, the WLI image of the VCE can also be compared with the SAVE images obtained from the HSI conversion algorithm. Figure S17 shows the SSIM for both the Olympus images and the VCE images. It can be seen that the Olympus images have a better SSIM rate with an average of 94.27% while the VCE has a comparatively lesser. Still, the average SSIM for VCE was found to be around 90%. This is because for NBI the CIEDE 2000 color calibration was possible for the traditional endoscope with the real NBI. After all, there was a reference real NBI image available. But for VCE no such reference is available. So, the same calibration that was done for the NBI images of the Olympus was done for VCE. Even though without any reference the algorithm achieved a SSIM of 90%. It can also be seen that the top three highest achieved SSIM values was from the VCE of 96%. From this, we can infer that the results of the study are accurate. In this study 50 randomly chosen VCE images were used for calculating the SSIM. However, by increasing the number of images the SSIM can be

significantly improved. Table S1 shows the SSIM comparison of twenty randomly chosen images in VCE and Olympus endoscope.

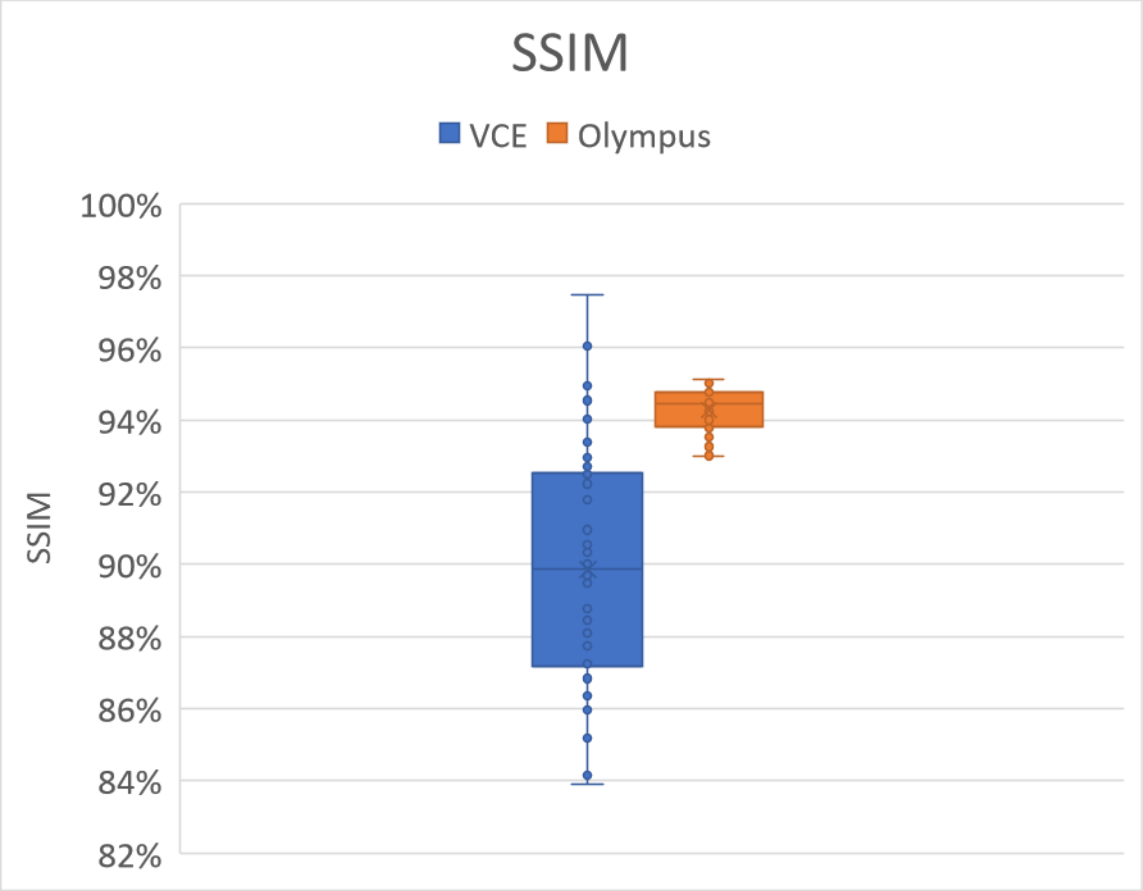

Figure S17. Comparison of SSIM between the simulated NBI images and the WLI images of VCE and Olympus

Table S1. SSIM of twenty randomly chosen images in VCE and Olympus endoscope.

Index	SSIM in VCE	SSIM in Olympus
1	0.925984794	0.94883467
2	0.944862636	0.948624384
3	0.928211385	0.94554478
4	0.966524653	0.937769205
5	0.92933774	0.941602499
6	0.958101429	0.945524249
7	0.939473779	0.935284698
8	0.936779292	0.933442268
9	0.872748906	0.938402164
10	0.873266671	0.936314054
11	0.891602213	0.934205211
12	0.944929655	0.949261138
13	0.805573811	0.941545835
14	0.90568673	0.946374234
15	0.83298981	0.945601764
16	0.912076221	0.932277471
17	0.936323837	0.930091206

18	0.792020729	0.939289678
19	0.936141641	0.932559509
20	0.903283862	0.935927389
Avg.	90.680%	93.992%

The second criterion that was used to evaluate the algorithm developed in this study was entropy. The entropy was also calculated similarly to the SSIM. The difference in entropy between the WLI images obtained from the Olympus endoscope is compared with the SAVE images simulated from the HSI conversion algorithm. In image processing, entropy might be used to classify textures, a certain texture might have a certain entropy as certain patterns repeat themselves in approximately certain ways. In the context of the paper low entropy means low disorder, low variance within the component. Therefore, lower the entropy better reproduction of the image is obtained. The difference in entropy between the WLI images obtained from VCE is compared with the SAVE images from the HSI-NBI conversion algorithm. Figure 18 shows the entropy difference in Olympus endoscope and VCE. As it can be seen from Table 4 the entropy difference in both the VCE and the Olympus endoscope have similar values. The average entropy difference in VCE was 1.17% while the average difference in the Olympus endoscope was 0.37%. However, in VCE the majority difference was found to be caused by only one image (image number 11). If we remove that image the entropy difference value is just 0.03% which is better than the Olympus endoscope. Table S2 shows the entropy comparison of the WLI and SAVE images in Olympus and VCE endoscope of twenty random images.

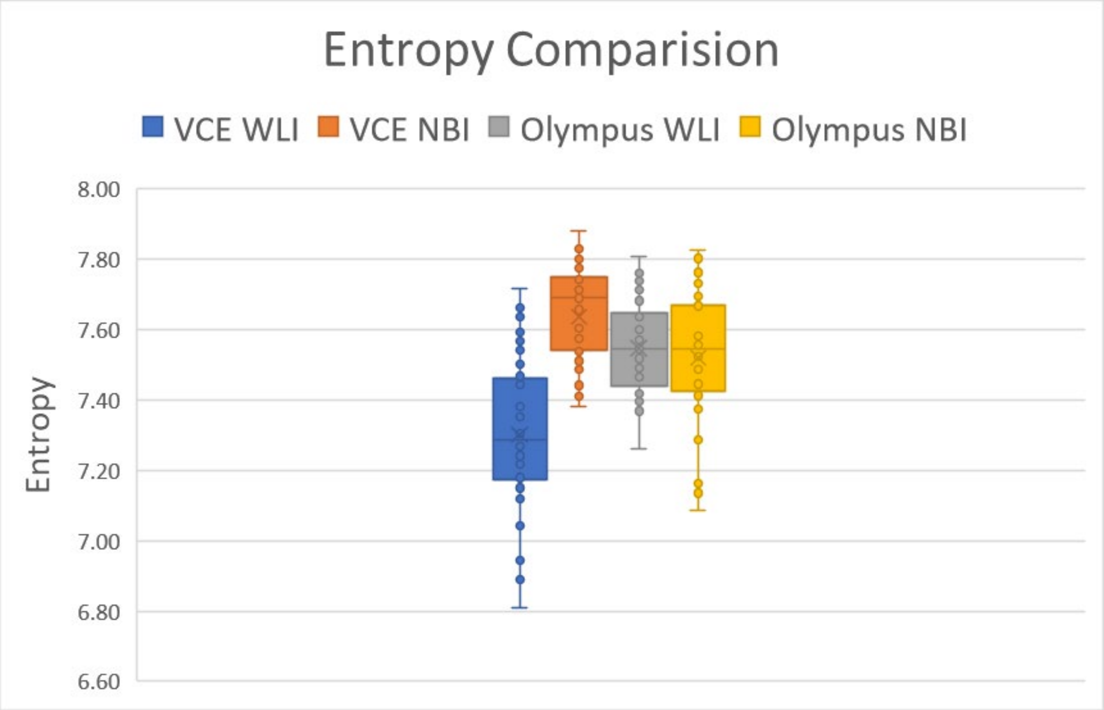

Figure S18. Comparison of entropy between the simulated NBI images and the WLI images. (a) Entropy for Olympus Endoscopy while (b) shows the entropy for the VCE camera.

Table S2. Entropy comparison of the WLI and NBI images in Olympus and VCE endoscope

Index	VCE		Endoscope		Difference in VCE	Difference in Olympus
	WLI	NBI	WLI	NBI		
1	7.04162	7.213345	7.60896	7.576404	0.024387	-0.004298
2	7.14502	7.220075	7.56380	7.494327	0.010504	-0.009271

3	7.37728	7.396152	7.65457	7.522734	0.002557	-0.017526
4	7.35948	7.278474	7.41264	7.283070	-0.011008	-0.017791
5	7.30720	7.363200	7.63545	7.680663	0.007663	0.005886
6	7.25352	7.350173	7.57114	7.546219	0.013325	-0.003167
7	6.88228	7.182308	7.53630	7.557263	0.043593	0.002773
8	7.25791	7.345877	7.71332	7.804702	0.012119	0.011708
9	6.95063	6.713150	7.56143	7.669351	-0.034167	0.014072
10	7.04094	7.134570	7.57816	7.676892	0.013297	0.012860
11	7.21094	6.575100	7.52565	7.372113	-0.088178	-0.020827
12	7.21771	7.219500	7.43596	7.422078	0.000247	-0.001871
13	7.58770	7.112978	7.57403	7.671311	-0.062566	0.012680
14	7.17281	7.184375	7.55415	7.667614	0.001612	0.014797
15	7.23312	7.210437	7.63470	7.693241	-0.003137	0.007608
16	7.14741	7.384973	7.71922	7.729203	0.033237	0.001291
17	7.28176	7.259479	7.75930	7.761126	-0.003061	0.000234
18	7.18062	6.801497	7.74727	7.801845	-0.052799	0.006994
19	7.26419	7.290983	7.74532	7.825175	0.003688	0.010205
20	7.59704	7.555495	7.736022	7.807877	-0.005469	0.009203
Avg	7.2254647	7.189607	7.61332	7.628160	-0.47%	0.19%

PSNR is typically utilized in the context of image compression algorithms as a parameter to evaluate the quality of the reproduced image. In a comparison of quality that is comparable to that of the SSIM, the PSNR values of twenty randomly selected WLI images and their SAVE equivalents are measured. The plot of the PSNR for each of the twenty images is displayed in Figure 19. The PSNR of the VCE images came in at an average of 28.0216 db, while the PSNR of the Olympus images was 27.8819 db. Table S3 shows the comparison of PSNR of the twenty randomly chosen images in Olympus and VCE.

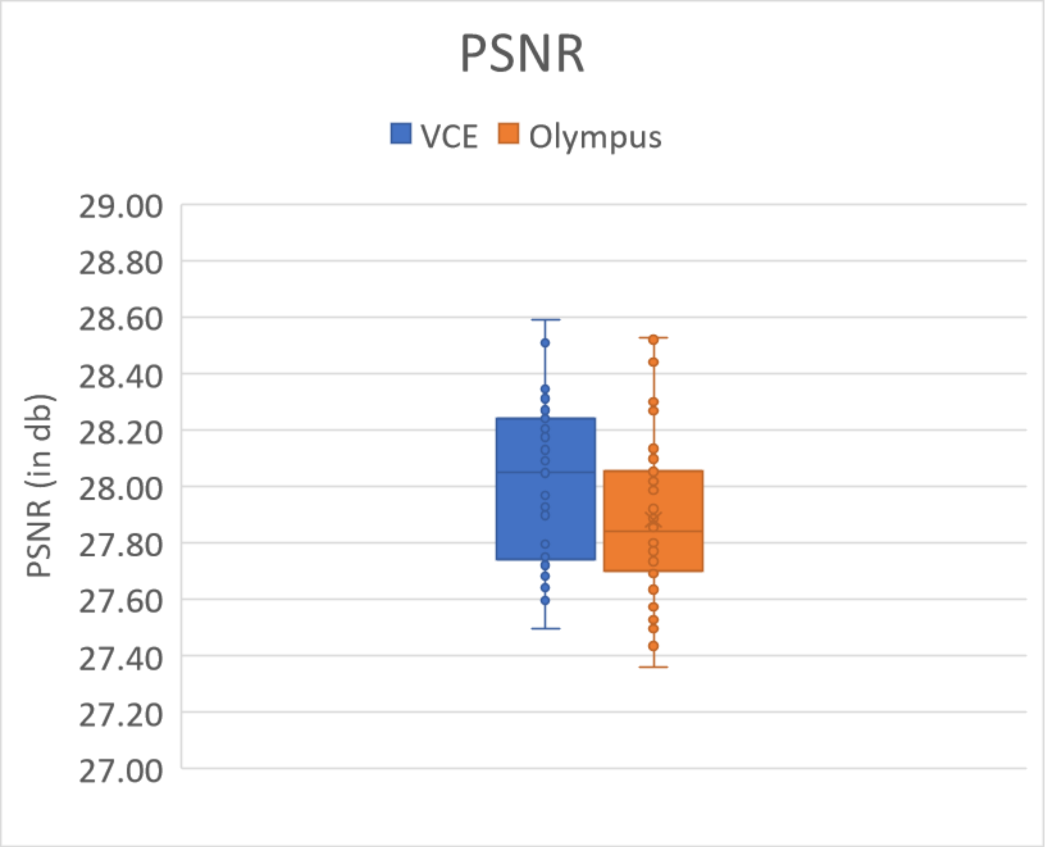

Figure S19. Comparison of PSNR of the twenty randomly chosen images in Olympus and VCE

Table S3. Comparison of PSNR of the twenty randomly chosen images in Olympus and VCE

Index	PSNR of Olympus images	PSNR of VCE images
1	27.5737493	28.51448883
2	27.4460567	27.72258326
3	27.46222796	27.66114567
4	27.36380762	27.49849716
5	27.82132106	27.67697859
6	27.49987858	28.1142441
7	27.78251246	27.90179575
8	27.71866018	27.6486571
9	27.78163772	27.65547302
10	27.63666623	28.0393251
11	27.58337722	28.20507339
12	27.70590967	27.99117518
13	27.53574671	28.26982928
14	27.68741185	27.9901207
15	27.74701762	28.26732897
16	27.81360865	27.7055983
17	27.87955844	27.90082759
18	27.71300104	28.50043856
19	27.73324857	27.68852332
20	28.02414942	27.68115621

Avg.	27.67547735	27.931663

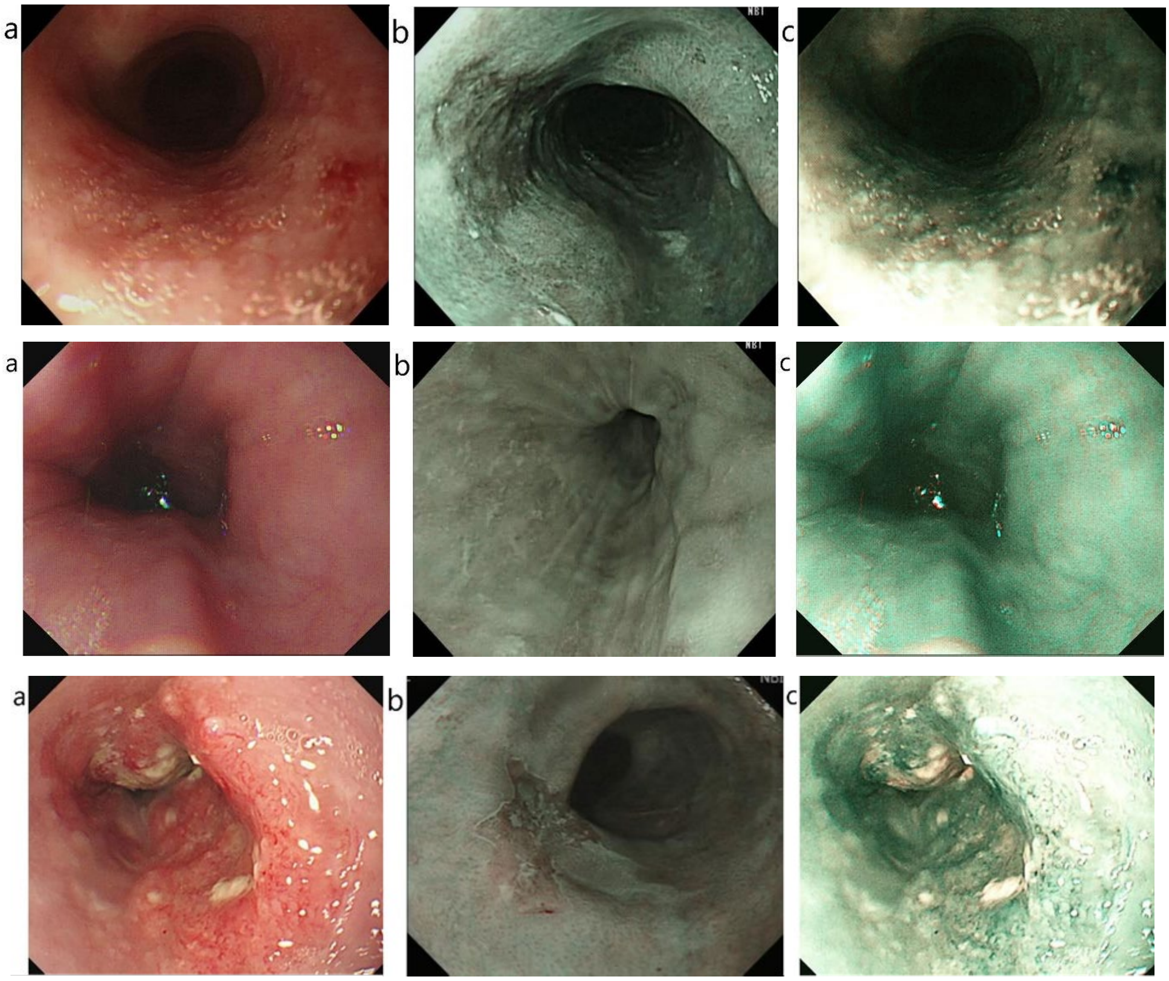

Figure S20. Endoscopic using three imaging techniques. (a) WLI, (b) NBI, (c) SAVE
